# Uptake of HIV testing among women of reproductive age in Tajikistan: An assessment of individual determinants

**DOI:** 10.5195/cajgh.2020.370

**Published:** 2020-03-31

**Authors:** Salima Kasymova

**Affiliations:** 1 Independent Researcher and Consultant

**Keywords:** HIV, HIV testing, Individual determinants, Tajikistan, Women

## Abstract

**Introduction:**

Over the past decade, the incidence of human immunodeficiency virus (HIV) infections in Tajikistan increased significantly, with women particularly vulnerable to acquiring HIV. This research assessed individual determinants associated with HIV testing among women of reproductive age.

**Methods:**

Secondary data analysis was done using data from 5,867 females aged 15-49 years. Chi-square test, t-test, and multivariate analysis were applied to find associations between women's socio-demographic characteristics, reproductive health variables, and HIV testing uptake.

**Results:**

Overall, only 26% (1,501) of women in the present research reported HIV testing in the past. Multiple regression indicated that HIV testing was significantly associated with participants' age (25-34 age group: OR 0.7, p ≤ 0.001; 35-49 age group: OR 0.2, p ≤ 0.001), education (OR 2.2, p ≤ 0.001), area of residence (OR 0.6, p ≤ 0.001), marital status (OR 2.4, p ≤ 0.001), HIV knowledge (OR 1.1, p ≤ 0.001), and pregnancy history (OR 6.7, p ≤ 0.001).

**Conclusion:**

Results of this research suggest that there is a need for culturally acceptable interventions, including outreach to increase the overall HIV testing rate among women in Tajikistan.

## Introduction

Tajikistan is one of the few countries in the world where the incidence of HIV infections is on the rise at a concerning rate. Between 2008 and 2017, the new HIV diagnosis rate increased in Tajikistan from 5.0 to 13.5 per 100,000 population. Particularly concerning is the growing number of new HIV cases attributable to heterosexual contact. The total number of people who acquired HIV via heterosexual contact increased between 2008 and 2017 by 640 cases (460%).^1^ Currently, about half of the country's population of nine million people is women. Evidence suggests that multiple structural and socio-cultural factors contribute to HIV risk for women in Tajikistan. Gender inequality, limited abilities to discuss fidelity and negotiate condom use, early marriage practices, and domestic violence may lead to increased HIV exposure. Women's vulnerability is also exacerbated by poverty, migration, and limited access to education and economic opportunities.^2^^–^^4^ The reported HIV prevalence rate in females increased in the country between 2008 and 2017 from 2.2 to 10.6 per 100,000 population, and AIDS diagnosis rate in females increased from 0.2 to 1.8 per 100,000 population. It is estimated that the cumulative total number of HIV cases among women in Tajikistan is 3,334.^1^

HIV testing is one of the most effective ways to halt the transmission of HIV. Pre- and post-test counseling sessions that occur during HIV testing services provide an opportunity for primary prevention.^5^ HIV testing also allows the provision of psychosocial support and links HIV positive individuals to antiretroviral therapy sites. Currently, HIV testing is mandatory for pregnant women in Tajikistan. Evidence suggests that HIV testing services are underutilized in the country and limited research explored this problem.^6^^,^^7^ Considering that Tajikistan has been experiencing one of the fastest growing HIV epidemics in the world, there is an urgent need to study the factors that govern individual choice to seek HIV testing services. Therefore, the goal of this study was to address this need by examining individual determinants associated with HIV testing among women in Tajikistan. Results of this research will potentially inform the development of future HIV prevention policies in Tajikistan aimed at reducing individual risk and vulnerability to HIV infection.

## Methods

### Data source

The current research used cross-sectional data from the Demographic and Health Survey (DHS)^8^, a nationally representative sample survey. The survey was conducted in Tajikistan in 2012 with an aim to collect data on maternal and child health, fertility and contraceptive use, domestic violence, tuberculosis, HIV, and other sexually transmitted infections (STIs) from women of reproductive age. The survey randomly selected 9,794 women aged 15-49 years from 6,674 households, of whom 9,656 were interviewed; the response rate for DHS was 99%.^9^

### Measures

For the HIV testing variable, participants' responses to the question “Have you ever been tested to see if you have the AIDS virus” were used; it was scored dichotomously (yes vs. no). Independent variables included several demographic and socioeconomic characteristics, as well as reproductive health variables, which were selected based on results of previous research.^10^^–^^13^ Demographic measures included respondents' age (15-24, 25-34, and 35-49), area of residence (urban vs. rural) and marital status (married/living with partner vs. unmarried/widowed/divorced/separated). Socioeconomic variables included education (no education/primary, secondary, and higher) and household income (low, middle, and high). HIV knowledge was assessed through an eight-item scale (α = 0.76) with response options of “yes”, “no”, and “I do not know”. HIV knowledge was defined as knowing two primary methods of HIV prevention (consistent condom use and staying faithful to one uninfected partner), rejecting four misconceptions about HIV transmission (HIV cannot be transmitted by mosquito bites, by sharing food, or by kissing, and a healthy-looking person can be infected with HIV) and knowing two facts about mother-to-child (i.e., vertical) HIV transmission (HIV can be transmitted during pregnancy and delivery). Women received one point for each correct answer, and all points summed to create an HIV knowledge score (range 0-8). Higher scores indicated higher levels of knowledge about HIV. Reproductive health variables included pregnancy history (had ever been pregnant vs. had never been pregnant) and had STIs in last 12 months (yes vs. no/do not know).

### Statistical analysis

As the first step of statistical analysis, descriptive approaches such as frequencies for binary and categorical variables, as well as means and standard deviations for continuous variables, were generated to describe the samples and understand the data distribution. Then, separate simple logistic regression equations with corresponding 95% confidence intervals (CIs) were conducted to identify the odds of having HIV testing for each of the independent variables: age, area of residence, marital status, education, income, HIV knowledge, pregnancy history, and had STIs in the last 12 months. Finally, a multivariate logistic regression model that included the HIV testing variable and all independent variables was estimated. A significance level of 0.05 was used during statistical analysis. The statistical analysis was conducted using STATA 13.

## Results

Of the 9,656 women surveyed, 3,587 reported not having heard of HIV and they were excluded from analysis. In addition, 202 cases with missing data were also excluded. Thus, the final sample for this research was 5,867 women. Sample characteristics are presented in Table 1. Age of the respondents ranged from 15 to 49 years, with a mean (SD) of 31 (9.8) years. Most women reported their marital status as married/living with partner (71%). The majority of respondents resided in rural areas (60%), had at least secondary education (97%), and were in the high wealth quintile (54%). Approximately 70% of the sample had been pregnant, and only 21 women (0.4%) indicated that they had an STI in the last 12 months. Lastly, HIV knowledge score ranged from 0 to 8, with participants' mean total HIV knowledge score at 4.9 (SD = 2.3), indicating moderate levels of overall HIV knowledge.

**Table 1.** Demographic and Socioeconomic Characteristics of Participants by HIV Testing History (n = 5,867)

Variable	Not tested n (%) Mean (SD)	Had been tested n (%) Mean (SD)	Total n (%) Mean (SD)	p-value
Age				
15–24	1,583 (36)	395 (26)	1,978 (34)	≤ 0.001^1^
25–34	1,107 (25)	645 (43)	1,752 (30)	
35–49	1,676 (38)	461 (31)	2,137 (36)	
Area of residence				
Urban	1,577 (36)	743 (50)	2,320 (40)	≤ 0.001^1^
Rural	2,789 (64)	758 (50)	3,547 (60)	
Marital status				
Married/living with partner	2,795 (64)	1,345 (90)	4,140 (71)	≤ 0.001^1^
Unmarried/widowed/divorced/separated	1,571 (36)	156 (10)	1,727 (29)	
Education				
No education/primaiy	116 (3)	36 (2)	152 (3)	≤ 0.001^1^
Secondary	3,278 (75)	913 (61)	4,191 (71)	
Higher	972 (22)	552 (37)	1,524 (26)	
Wealth mdex				
Low	1,374 (31)	337 (22)	1,711 (29)	≤ 0.001^1^
Middle	761 (17)	226 (15)	987 (17)	
High	2,231 (51)	938 (62)	3,169 (54)		
HIV knowledge	4.7 (2.4)	5.4 (2.1)	4.9 (2.3)	≤ 0.001^2^
Pregnancy history				
Had ever been pregnant	2,744 (63)	1,359 (91)	4,103 (70)	≤ 0.001^1^
Had never been pregnant	1,622 (37)	142 (9)	1,764 (30)	
Had STI in last 12 months				
Yes	14 (0.3)	7 (0.5)	21 (0.4)	0.415^1^
No/Do not know	4,352 (99.7)	1,494 (99.5)	5,846 (99.6)	

1 Chi-Square test;

2 t-test.

Overall, 1,501 (26%) of respondents reported HIV testing in the past. Table 2 presents unadjusted and adjusted estimates from logistic regression models predicting the odds of having HIV testing for each of the independent variables with corresponding 95% CIs. The odds of being HIV tested were significantly lower among rural women, unmarried/widowed/divorced/separated women, and those with lower education and income. In addition, women with HIV testing history had significantly higher HIV knowledge scores as compared with their counterparts who had not been tested. Lastly, the likelihood of HIV testing was correlated with respondents' age and pregnancy history.

**Table 2.** Unadjusted and Adjusted Estimates from Logistic Regression Models Predicting HIV Testing among Women (n = 5,867)

Variable	Unadjusted		Adjusted	
	OR	95% CI	OR	95% CI
Age				
15–24 (ref.)	1.0		1.0	
25–34	2.3***	2.0–2.9	0.7***	0.6–0.9
35–49	1.1	0.9–1.3	0.2***	0.2–0.3
Area of residence				
Urban (ref.)	1.0		1.0	
Rural	0.6***	0.5–0.6	0.6***	0.5–0.7
Marital status				
Unmarried/widowed/divorced/separated (ref.)	1.0		1.0	
Married/living with partner	4.8***	4.1–5.8	2.4***	1.9–3.0
Education				
No education/primary (ref.)	1.0		1.0	
Secondary	0.9	0.6–1.3	1.0	0.7–1.6
Higher	1.8**	1.2–2.7	2.2***	1.4–3.4
Household wealth				
Low (ref.)	1.0		1.0	
Middle	1.2*	1.0–1.5	1.0	0.8–1.2
High	1.7***	1.5–2.0	1.0	0.9–1.3
HIV knowledge	1.2***	1.1–1.2	1.1***	1.1–1.2
Pregnancy history				
Had never been pregnant (ref.)	1.0		1.0	
Had ever been pregnant	5.7***	4.7–6.8	6.7***	5.1–8.8
Had STI in last 12 months				
No/Do not know (ref.)	1.0		1.0	
Yes	1.5	0.6–3.6	0.7	0.3–1.9

* p ≤ 0.05;

** p ≤ 0.01;

*** p ≤ 0.001

In the adjusted logistic regression model, six significant predictors of HIV testing: age, area of residence, marital status, education, HIV knowledge, and pregnancy history. The strongest predictor of HIV testing was pregnancy history; women who had ever been pregnant were almost seven times (OR 6.7, 95% CI 5.1-8.8, p ≤ 0.001) more likely to report that they had been tested for HIV in the past. In addition, married women and women who lived with a partner were more than twice (OR 2.4, 95% CI 1.9-3.0, p ≤ 0.001) as likely to report HIV testing uptake as compared to unmarried women and women who were divorced, separated, and widowed. The odds of ever testing for HIV were also significantly higher among urban women (OR 0.6, 95% CI 0.5-0.7, p ≤ 0.001), women with higher education (OR 2.2, 95% CI 1.4-3.4, p ≤ 0.001), and women with higher levels of HIV knowledge (OR 1.1, 95% CI 1.1-1.2, p ≤ 0.001). Lastly, the odds of ever being tested for HIV decreased with women's age (for 25-34 age group: OR 0.7, 95% CI 0.6-0.9, p ≤ 0.001 and for 35-49 age group: OR 0.2, 95% CI 0.2-0.3, p ≤ 0.001).

## Discussion

This research was motivated by the need to learn more about uptake of HIV testing among women of reproductive age in Tajikistan. Results of this research suggested that within the sample, approximately three out of four women aged 15-49 years had never been tested for HIV. Respondents' age, area of residence, marital status, education, HIV knowledge, and pregnancy history were significant predictors of undergoing HIV testing.

Taking into consideration that according to the national legislations, pregnant women are subject to mandatory HIV testing, it is not surprising that pregnancy history was the strongest HIV testing predictor. This result corroborates the idea that antenatal care is an important gateway to HIV testing among women in Tajikistan. On the other hand, it raised the concern of access to HIV testing for women who had never been pregnant and older women who are less likely to seek antenatal care services.

Findings also indicate that urban women were more likely to have been tested for HIV than rural women. Lower HIV testing uptake among rural women may be related to lower accessibility to HIV testing services in rural areas of Tajikistan.^3^ This finding could also be related to the geography of the HIV epidemic, since the prevalence of HIV in Tajikistan is higher in urban areas.^4^

Consistent with the results of previous research^10^, marital status was one of the significant determinants of HIV testing. This finding may reflect the fact that the HIV testing promotion strategies fail to reach women who are unmarried, widowed, divorced, and separated. In addition, as previously documented^11^^–^^13^, higher level knowledge about HIV was associated with higher odds of HIV testing. This finding could be an indication that women with more accurate HIV knowledge acknowledge their risk for contracting HIV and seek opportunities to know their HIV serostatus.

Lastly, the unadjusted logistic regression models indicate HIV testing uptake increased with women's wealth status. However, in the adjusted regression model, the relationship between these variables was positive but not significant. This finding contradicts results of studies conducted in other developing countries.^10^^,^^11^^,^^14^^,^^15^ This result may suggest that epidemiological and contextual factors play a more significant role for seeking HIV testing services than individual determinants. Nevertheless, further studies are needed to understand how individual, epidemiological, and contextual factors influence HIV testing uptake in Tajikistan.

The following limitations should be considered in interpretation of these research findings. Due to the cross-sectional design of this research, it was not possible to determine causal inferences about relationships among HIV testing and independent variables. Another limitation is that the research sample consisted of women aged 15-49 years and more than half of them were from households with high income, which makes it difficult to generalize the findings to females of other age and income groups. In addition, DHS data were self-reported, which may have introduced social desirability and recall bias. This research focused on how individual characteristics affect uptake of HIV testing; however, available literature suggests that community-level determinants may play a significant role in HIV testing uptake. For example, previous research^14^ has shown that women from more educated and higher income communities had higher odds of HIV testing. Moreover, Kuehne and colleagues^12^ found that respondents who lived in the communities that discussed HIV were twice as likely to have been tested for HIV. Hence, future research should consider the incorporation of broader arrays of variables, including community-level determinants, which can affect women's behaviors to seek HIV testing services. Finally, for this research, data collected in 2012 was used, and its applicability today may be a matter of concern. Therefore, when 2017 DHS data becomes available, future research should replicate the analysis to determine any changes in factors that play a significant role in HIV testing behaviors among women of Tajikistan.

In view of results of this research, to increase HIV testing uptake among women in Tajikistan, several policies and interventions can be considered. There is a need for innovative and culturally acceptable interventions that include outreach efforts targeting adolescent females; older women; women from rural areas; unmarried, widowed, divorced, and separated women; as well as women who had never been pregnant. Special attention should also be given to the promotion of HIV testing among pregnant women. Every year, approximately 200,000 women in Tajikistan become pregnant^3^ with a high proportion never receiving antenatal care. According to the 2012 Tajikistan DHS report^9^, 21% of women did not receive any antenatal care for their most recent pregnancy. Moreover, between 2010 and 2013, the number of new HIV infections diagnosed among pregnant women increased from 53 to 112 cases.^4^ Available literature suggests that home-based HIV testing and mobile-clinic HIV testing can contribute to improving coverage and accessibility to HIV testing.^16^^,^^17^ In addition, provider-initiated testing can help to increase the overall HIV testing rate^13^, and it should be promoted in the future. Finally, previous research reported that many women do not seek HIV testing services because their brothers or mothers-in-law forbid them to do so.^3^ Moreover, available data suggests that spouses rarely discuss HIV testing between each other.^18^ Asking a husband/partner to test for HIV is particularly difficult for a woman due to power dynamics within the relationship. Therefore, HIV prevention interventions should focus on increasing general knowledge about HIV, addressing community norms about HIV testing, and eliminating HIV-related stigma. In addition, communication with a spouse/partner should be included in HIV prevention efforts.

To conclude, the findings of this research add to the scant literature on determinants of HIV testing uptake among women aged 15-49 years in Tajikistan. Results of this research suggest that there is a need for culturally acceptable interventions, including outreach to increase the overall HIV testing rate among women in Tajikistan.
